# Association between Higher Serum Cortisol Levels and Decreased Insulin Secretion in a General Population

**DOI:** 10.1371/journal.pone.0166077

**Published:** 2016-11-18

**Authors:** Aya Kamba, Makoto Daimon, Hiroshi Murakami, Hideyuki Otaka, Kota Matsuki, Eri Sato, Jutaro Tanabe, Shinobu Takayasu, Yuki Matsuhashi, Miyuki Yanagimachi, Ken Terui, Kazunori Kageyama, Itoyo Tokuda, Ippei Takahashi, Shigeyuki Nakaji

**Affiliations:** 1 Department of Endocrinology and Metabolism, Hirosaki University Graduate School of Medicine, Hirosaki, Aomori, Japan; 2 Department of Social Medicine, Hirosaki University Graduate School of Medicine, Hirosaki, Aomori, Japan; Jichi Medical University, JAPAN

## Abstract

Glucocorticoids (GCs) are well known to induce insulin resistance. However, the effect of GCs on insulin secretion has not been well characterized under physiological conditions in human. We here evaluated the effect of GCs on insulin secretion/ß-cell function precisely in a physiological condition. A population-based study of 1,071 Japanese individuals enrolled in the 2014 Iwaki study (390 men, 681 women; aged 54.1 ± 15.1 years), those excluded individuals taking medication for diabetes or steroid treatment, were enrolled in the present study. Association between serum cortisol levels and insulin resistance/secretion assessed by homeostasis model assessment using fasting blood glucose and insulin levels (HOMA-R and HOMA-ß, respectively) were examined. Univariate linear regression analyses showed correlation of serum cortisol levels with HOMA-ß (ß = -0.134, p <0.001) but not with HOMA-R (ß = 0.042, p = 0.172). Adjustments for age, gender, and the multiple clinical characteristics correlated with HOMA indices showed similar results (HOMA-ß: ß = -0.062, p = 0.025; HOMA-R: ß = -0.023, p = 0.394). The correlation between serum cortisol levels and HOMA-ß remained significant after adjustment for HOMA- R (ß = -0.057, p = 0.034). When subjects were tertiled based on serum cortisol levels, the highest tertile was at greater risk of decreased insulin secretion (defined as lower one third of HOMA-ß (≤70)) than the lowest tertile, after adjustment for multiple factors including HOMA- R (odds ratio 1.26, 95% confidence interval 1.03–1.54). In conclusion, higher serum cortisol levels are significantly associated with decreased insulin secretion in the physiological cortisol range in a Japanese population.

## Introduction

Type 2 diabetes (hereafter diabetes) is a heterogeneous disorder of glucose metabolism characterized by both insulin resistance and pancreatic ß-cell dysfunction. Glucocorticoids (GCs) are known to be among the various conditions and factors involved in the pathophysiology of diabetes, with an excess of GC, from GC administration or in pathological conditions such as Cushing syndrome, leading to diabetes [1–7]. GCs promote gluconeogenesis by inducing expression of gluconeogenic genes in the liver; and by suppressing glucose uptake in skeletal muscle and adipocytes (inhibiting translocation of glucose transporter GLUT 4 to the cell surface) [5–9], which induces insulin resistance. Therefore, insulin resistance is likely to be an important mechanism by which an excess GC leads to diabetes.

Less is known about whether an excess of GC affects pancreatic ß-cell function and insulin secretion. Generally, insulin secretion increases compensatory along with increases in insulin resistance to maintain plasma glucose levels as normal as possible. If this compensatory increase in insulin secretion fails, plasma glucose levels rise leading to diabetes. Indeed, increased serum insulin levels were observed in most studies with Cushing syndrome or GC administration [1, 10–15]. However, these facts cannot be simply explained that GC increases ß-cell function. In vitro experiments with cultured ß-cells showed that GC suppress insulin secretion [16]. Furthermore, in vivo experiments with transgenic mice that over express GC receptor specifically in ß-cells showed decreased insulin secretion during a glucose load [17, 18]. GCs thus appear to suppress ß-cell function directly, suggesting that higher GC levels increase the risk of impaired glucose metabolism independent of their induction of insulin resistance. Since, as described previously, the effects of GCs on ß-cell function observed in subjects with pathologically high serum cortisol levels are a mixture of direct and indirect (or compensatory) effects, the direct effects of GCs on insulin secretion are yet to be elucidated, especially in humans.

To properly evaluate the effects of GCs on ß-cell function in humans, we examined the relationship between serum cortisol levels and ß-cell function, represented by homeostatic model assessment (HOMA) indices (ß and R for insulin secretion and resistance, respectively), in a general Japanese population with adjustment for insulin resistance. To our knowledge, this relationship has not yet been properly evaluated in a general population with physiological serum cortisol levels and no obvious insulin resistance.

## Subjects and Methods

### Subjects

Our subjects were recruited from the Iwaki study, a health promotion study of Japanese people over 20 years of age that aims to prevent lifestyle-related diseases and prolong lifespans. The study is conducted annually in the Iwaki area of the city of Hirosaki in Aomori Prefecture located in northern Japan [19, 20]. Of the 1167 individuals enrolled in the Iwaki study in 2014, the following individuals were excluded from our study: 2 on steroid treatment (which substantially affects serum cortisol levels), 5 with incomplete clinical data, and 58 on medication for diabetes, which affects insulin sensitivity and resistance substantially. HOMA-ß can be calculated only when fasting blood glucose levels are more than 64 mg/dl. HOMA-R is well correlated with the gold standard index of insulin resistance, M-value, evaluated by using euglycemic hyperinsulinemic glucose technique [21]. However, such correlation has been reported to become less accurate when fasting blood glucose levels are more than 140 mg/dl probably because of disruption of homeostasis between glucose and insulin [22, 23]. Therefore, we excluded also 31 subjects with fasting blood glucose levels lower than 63 mg/dl or more than 140 mg/dl, to evaluate HOMA indices precisely. After these exclusions, 1,071 individuals (390 men, 681 women) aged 54.1 ± 15.1 years were included in our study.

This study was approved by the Ethics Committee of the Hirosaki University School of Medicine (No. 2014–014 and 2014–015), and written informed consent was obtained from all participants.

### Characteristics measured

Blood samples were collected in the morning from peripheral veins of participants under fasting conditions in a supine position. Serum cortisol concentrations were determined using a chemiluminescent enzyme immunoassay, according to the manufacturer’s instructions (Access Cortisol Kit; Beckman Coulter, Inc., Tokyo, Japan). The following clinical characteristics were also measured: height, body weight, body mass index, percent body fat (fat), fasting blood glucose, fasting serum insulin levels, glycated hemoglobin (HbA1c), systolic blood pressure, diastolic blood pressure, total serum levels of total cholesterol, triglyceride, high-density lipoprotein-cholesterol, uric acid, urea nitrogen, creatinine, brain natriuretic peptide (BNP), and adiponectin. Fat was measured by the bioelectricity impedance method using a Tanita MC-190 body composition analyzer (Tanita Corp., Tokyo, Japan). HbA1c (%) is expressed as the National Glycohemoglobin Standardization Program value. Insulin resistance and secretion were assessed by homeostasis model assessment using fasting blood glucose and insulin levels (HOMA-R and HOMA-ß, respectively). Diabetes was defined according to the 2010 Japan Diabetes Society criteria (fasting blood glucose levels ≥ 126 mg/dL) [24]. In subjects whose fasting blood glucose levels were not measured, diabetes was defined as HbA1c levels ≥ 6.5%. Those on medication for diabetes were also defined as having diabetes. None of the subjects had diagnosed type 1 diabetes. Hypertension was defined as blood pressure ≥ 140/90 mmHg or taking treatment for hypertension. Hyperlipidemia was defined as total cholesterol ≥ 220 mg/dL, triglyceride ≥ 150 mg/dL or taking treatment for hyperlipidemia. Alcohol intake (current or non-drinker) and smoking habits (never, past or current) were determined from questionnaires.

### Statistical methods

Clinical characteristics are given as means ± SD. The statistical significance of the difference in characteristics values between two groups (parametric) and case-control associations between groups (nonparametric) were assessed by analysis of variance and χ^2^ tests, respectively. Correlations between HOMA indices and clinical characteristics, including serum cortisol levels, were assessed by linear regression analyses. For statistical analyses, HOMA indices, serum cortisol levels, BNP and adiponectin were log-transformed (log10) to approximate a normal distribution. Risk of higher serum cortisol for decreased insulin secretion was calculated by multiple logistic regression analysis with adjustment for factors found to be associated with insulin secretion by univariate regression analysis. A value of p<0.05 was accepted as statistically significant. All analyses were done using SPSS version 23.0 (IBM Japan, Tokyo, Japan).

## Results

### Clinical characteristics of the study subjects

The clinical characteristics of subjects by gender are shown in S1 Table. Mean ages were 52.2 ± 14.9 years for men and 55.3 ± 15.1 years for women. Most clinical characteristics were significantly different between men and women, as serum cortisol levels were significantly higher in men than women (10.3 ± 3.5 *vs*. 8.6 ± 3.3 μg/dL).

Prevalence of hypertension, hyperlipidemia and diabetes for men and women were 48.0% and 41.0%, 42.6% and 42.1%, and 4.9% and 3.5%, respectively. The prevalence of hypertension is similar to the 2010 national values for men and women aged 30 to 69 years reported by the Japanese government (50.8% and 33.7%, respectively) [25]. There are no reported national values for hyperlipidemia prevalence (using the same definition as this study), but our observed prevalence is similar to that reported in the other areas of Japan [26–29]. We excluded individuals with diabetes from the present study (33 men, 33 women), but with these individuals the prevalence of diabetes in men and women in our original sample (11.7% and 7.9%, respectively) was similar to the Japanese national values (15.4% and 7.1%, respectively) [25]. More men drank alcohol and were current smokers than women (73.6% *vs*. 31.9%, and 31.0% *vs*. 8.2%, respectively).

### Correlation between serum cortisol levels and HOMA indices (or insulin resistance and secretion)

Correlations between clinical characteristics and HOMA-R (representing insulin resistance) are shown in Table 1. Many clinical characteristics such as gender, age, fat percent, and serum levels of total cholesterol, albumin, BNP and adiponectin were found to be correlated with HOMA-R, even with adjustments for multiple factors. However, serum cortisol levels were not correlated with HOMA-R (Univariate: ß = -0.042, p = 0.172; Multivariate: ß = -0.023, p = 0.394).

**Table 1:** Facotrs correlated with HOMA-R.

	Univariate		Multivariate	
Characteristics	ß	p	ß	p
**Gender (M/F)**	**0.077**	0.012*	**-0.116**	0.013*
**Age (yr)**	**0.071**	0.020*	**0.083**	0.014*
**Height (cm)**	**-0.067**	0.029*	**-**	**-**
**Body weight (kg)**	**0.337**	<0.001**	**-**	**-**
**Body mass index (kg/m**^**2**^**)**	**0.497**	<0.001**	**-**	**-**
**Fat (%)**	**0.465**	<0.001**	**0.521**	<0.001**
**Cortisol (μg/dl)**	**-0.042**	**0.172**	**-0.023**	**0.394**
**Fasting plasma glucose (mg/dl)**	**0.457**	<0.001**	**-**	**-**
**HbA1c (%)**	**0.281**	<0.001**	**-**	**-**
**Fasting serum insulin: IRI (μU/ml)**	**0.903**	<0.001**	**-**	**-**
**HOMA-ß**	**0.307**	<0.001**	**-**	**-**
**Systolic blood pressure (mmHg)**	**0.161**	<0.001**	**0.049**	**0.094**
**Diastolic blood pressure (mmHg)**	**0.168**	<0.001**	**-**	**-**
**Total cholesterol (mg/dl)**	**0.131**	<0.001**	**-0.014**	**0.602**
**Triglyceride (mg/dl)**	**0.224**	<0.001**	**0.111**	<0.001**
**HDL cholesterol (mg/dl)**	**-0.234**	<0.001**	**-**	**-**
**Serum albumin (g/dl)**	**0.138**	<0.001**	**0.136**	<0.001**
**Serum uric Acid (mg/dl)**	**0.125**	<0.001**	**0.067**	**0.053**
**Serum urea Nitrogen (mg/dl)**	**0.045**	**0.14**	**-**	**-**
**Serum creatinin (mg/dl)**	**0.05**	**0.103**	**-**	**-**
**BNP (pg/ml)**	**-0.114**	<0.001**	**-0.107**	0.001**
**Adiponectin (mg/dl)**	**-0.186**	<0.001**	**-0.085**	0.010*
**Hypertension: n (%)**	**0.219**	<0.001**	**-**	**-**
**Hyperlipidemia: n (%)**	**0.227**	<0.001**	**-**	**-**
**Diabetes: n (%)**	**0.14**	<0.001**	**-**	**-**
**Drinking alcohol: n (%)**	**-0.14**	<0.001**	**-0.077**	0.007**
**Smoking (Never/ Past/ Current):n**	**-0.083**	0.007**	**-0.031**	**0.308**

P<0.05 and <0.01 are indicated by * and **, respectively. Data are mean±SD or number of subjects (%).

Correlations between clinical characteristics and HOMA-ß (representing insulin secretion) were also examined (Table 2). Univariate regression analyses revealed correlations between HOMA-ß and many clinical characteristics including serum cortisol levels (ß = -0.134, p<0.001). Further, the correlation between HOMA-ß and serum cortisol levels remained significant after adjustment for multiple factors (sex, age, fat, systolic blood pressure, serum levels of total cholesterol, triglyceride, albumin, urea nitrogen, BNP and adiponectin and drinking alcohol) (ß = -0.062, p = 0.025). Since there is a compensatory increase in insulin secretion under conditions of insulin resistance, we further examined the correlation with adjustment for HOMA-R, which did not change its significance (ß = -0.057, p = 0.034). Analyses of subjects without insulin resistance (23 subjects with HOMA-R ≥ 2.5 were excluded) gave further evidence of the correlation (ß = -0.059, p = 0.030).

**Table 2:** Facotrs correlated with HOMA-ß.

	Univariate		Multivariate	
Characteristics	ß	p	ß	p
**Gender (M/F)**	**0.137**	<0.001**	**0.024**	**0.574**
**Age (yr)**	**-0.415**	<0.001**	**-0.363**	<0.001**
**Height (cm)**	**0.035**	**0.259**	**-**	**-**
**Body weight (kg)**	**0.127**	<0.001**	**-**	**-**
**Body mass index (kg/m**^**2**^**)**	**0.141**	<0.001**	**-**	**-**
**Fat (%)**	**0.211**	<0.001**	**0.246**	<0.001**
**Cortisol (μg/dl)**	**-0.134**	<0.001**	**-0.062**	0.025*
**Fasting plasma glucose (mg/dl)**	**-0.628**	<0.001**	**-**	**-**
**HbA1c (%)**	**-0.337**	<0.001**	**-**	**-**
**Fasting serum insulin: IRI (μU/ml)**	**0.45**	<0.001**	**-**	**-**
**HOMA-R**	**0.307**	<0.001**	**-**	**-**
**Systolic blood pressure (mmHg)**	**-0.192**	<0.001**	**-0.042**	**0.163**
**Diastolic blood pressure (mmHg)**	**-0.086**	0.005**	**-**	**-**
**Total cholesterol (mg/dl)**	**-0.067**	0.029*	**-0.062**	0.028*
**Triglyceride (mg/dl)**	**0.112**	<0.001**	**0.12**	<0.001**
**HDL cholesterol (mg/dl)**	**0.142**	<0.001**	**-**	**-**
**Serum albumin (g/dl)**	**0.107**	<0.001**	**-0.003**	**0.924**
**Serum uric Acid (mg/dl)**	**-0.009**	**0.778**	**-**	**-**
**Serum urea Nitrogen (mg/dl)**	**-0.283**	<0.001**	**-0.083**	**0.006**
**Serum creatinin (mg/dl)**	**-0.05**	**0.101**	**-**	**-**
**BNP (pg/ml)**	**-0.245**	<0.001**	**-0.068**	0.037*
**Adiponectin (mg/dl)**	**-0.163**	<0.001**	**-0.017**	**0.613**
**Hypertension: n (%)**	**-0.177**	<0.001**	**-**	**-**
**Hyperlipidemia: n (%)**	**0.003**	**0.932**	**-**	**-**
**Diabetes: n (%)**	**-0.192**	<0.001**	**-**	**-**
**Drinking alcohol: n (%)**	**-0.128**	<0.001**	**-0.114**	<0.001**
**Smoking (Never/ Past/ Current):n**	**0.005**	**0.863**	**-**	**-**

P<0.05 and <0.01 are indicated by * and **, respectively. Data are mean±SD or number of subjects (%).

The correlations between serum cortisol levels and HOMA indices are summarized in Table 3. Age- and sex-adjusted correlation coefficients were included. Serum cortisol levels were correlated with insulin secretion, but not with insulin resistance.

**Table 3:** Correlation of serum cortisol levels with HOMA indices.

	Univariate		Age and gender adjusted		Multiple factors adjusted	
	ß	p	ß	p	ß	p
**HOMA-R**	**-0.042**	**0.172**	**-0.028**	**0.369**	**-0.023**	**0.394**
**HOMA-ß**	**-0.134**	<0.001**	**-0.081**	0.004**	**-0.062**	0.025**

P<0.05 and <0.01 are indicated by * and **, respectively. Multiple factors: HOMA-R: Age, Gender, Fat, Systolic blood pressure, Total cholesterol, Triglyceride, Serum albumin, Serum uric acid, BNP, adiponectin, Drinking alcohol, Smoking. HOMA-ß: Age, Gender, Fat, Systolic blood pressure, Total cholesterol, Triglyceride, Serum albumin, Serum urea nitrogen, BNP, adiponectin, Drinking alcohol.

### Association of higher serum cortisol levels and decreased insulin secretion

**Fig 1:**
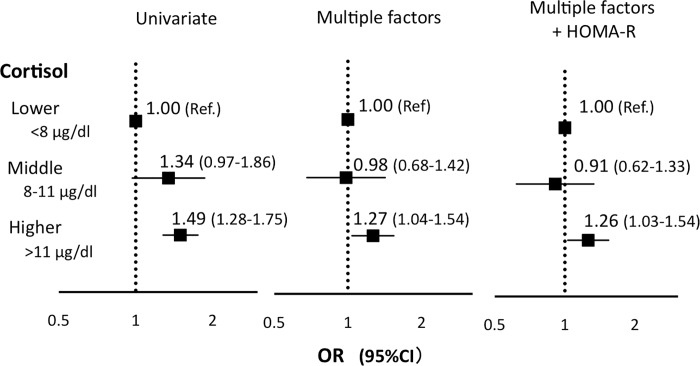
Risk for decreased insulin secretion. Odds ratio (OR)s with 95%confidence interval (CI) are shown. Multiple factors: Sex, Age, Fat, Systolic blood pressure, Total cholesterol, Triglyceride, Serum albumin, Serum urea nitrogen, BNP, adiponectin, Drinking alcohol. Ref: reference.

To further evaluate the relationship between serum cortisol levels and insulin secretion, subjects were stratified into tertiles based on their serum cortisol levels (higher >11 μg/dL, middle 8–11 μg/dL, lower <8 μg/dL). We then evaluated the risks of these tertiles for decreased insulin secretion, which we designated as the lower one third of HOMA-ß (≤ 70) (Fig 1). Higher serum cortisol levels were a significant risk for decreased insulin secretion (odds ratio (OR) 1.49, 95% confidence interval (CI) 1.28–1.75). The risk remained significant after adjustment for sex and age (OR 1.34, 95% CI 1.12–1.61) and multiple other factors (sex, age, fat, systolic blood pressure, serum levels of total cholesterol, triglyceride, albumin, urea nitrogen, BNP and adiponectin and drinking alcohol) (OR 1.27, 95% CI 1.04–1.54). Further adjustment for HOMA-R did not change the significance (OR 1.26, 95% CI 1.03–1.54). Together, these results indicate that higher serum cortisol levels are a significant risk for decreased insulin secretion in this general Japanese population.

## Discussion

In this cross-sectional study of a general Japanese population, we found that seruμ cortisol levels in a physiological range are significantly correlated with HOMA-ß but not with HOMA-R. The negative correlation between serum cortisol levels and HOMA-ß observed here appears contrary to previous studies on subjects with pathologically high serum cortisol levels. In the latter, a positive correlation was found between serum cortisol levels and HOMA-ß because of a compensatory increase in serum insulin levels with insulin resistance [12–15]. However, this positive correlation may not reflect the true effect of GCs on insulin secretion. Using a general population, we revealed effects of GC on HOMA-ß (or ß-cell function) independent of insulin resistance, because our subjects had serum cortisol levels in a physiological range and no/mild insulin resistance. Statistical adjustments for insulin resistance or HOMA-R further confirmed the negative correlation. These findings indicate that physiological levels of GC suppress ß-cell function but do not induce insulin resistance. Therefore, although pathological levels of GCs have been well documented to be positively correlated with insulin resistance together with compensatory insulin secretion, suppression of ß-cell function may be a more fundamental pathophysiology of GCs on glucose metabolism than insulin resistance. Namely, although it is hypothetical, GCs suppress ß-cell insulin secretion in a physiological range, and when GC levels increase to pathological levels, insulin resistance develops and the ß-cell dysfunction and insulin resistance imposed by GCs together lead to impaired glucose metabolism or diabetes. In this context, higher serum cortisol levels seem to be a risk factor for impaired ß-cell function or diabetes, whether they are in the pathological range or the physiological range. Further studies are required to elucidate the mechanisms through which GCs may suppress ß-cell function.

We recognize that correlation does not imply a cause and effect relationship, by the nature of the analysis. Therefore, the observed negative correlation between serum cortisol levels and HOMA-ß per se may not mean that GCs necessarily suppress ß-cell function. However, several basic experiments have revealed that GCs suppress ß-cell function directly [5, 6, 16–18]; thus, the observed correlation may be a cause and effect relationship. However, another explanation can be made for this result. Although serum cortisol levels are responsible for the GC receptor-mediated effects that lead to diabetes [4, 30], serum cortisol levels can also represent hypothalamus-pituitary-adrenal axis (HPA axis) activity, and HPA activation is known to result in diabetes. The effects of the HPA axis have been well documented in humans with pathologically high HPA activation levels, as seen in Cushing syndrome [1, 2]. The HPA axis is a major stress response mediator and acts in conjunction with another major mediator, the autonomic nervous system (ANS). The HPA axis and ANS are closely coupled, and are often activated in parallel [31, 32]. Therefore, HPA axis activation can occur in parallel with activation of the ANS [33], and serum cortisol levels may also represent ANS activity. The ß-cell dysfunction of transgenic mice overexpressing the GC receptor specifically in ß-cells was prevented by incubation of the islets with benextramine, a selective αlfa2-adrenergic receptor agonist [6]. Furthermore, ß-cell dysfunction evaluated using an insulinogenic index in mice treated with hydrocortisone was reported to be prevented by chlorisondamine (a ganglionic blocker) or phentolamine [34]. These findings indicate that higher serum cortisol levels represent, at least in part, an activation of adrenergic signals, and are thus associated with decreased ß-cell dysfunction. The direct effect of GC on ß-cells in vivo may be difficult to study, because the systemic effects of GCs mask these direct effects. Although GCs appear to suppress ß-cell function through GC receptor mediated mechanisms and/or an activation of adrenergic signals, the details of the mechanisms are yet to be elucidated.

Our study has both strengths and limitations. For strengths, statistical adjustments were made for multiple factors that could confound the results, and a relatively large population-based/general sample of individuals was used. Most importantly, we used a general population, and can thus evaluate the effects of GCs on insulin secretion without the influence of compensatory increases in insulin secretion. For limitations, the subjects were participants in a health promotion study rather than an ordinary health check-up study, and may be more invested in keeping themselves healthy compared with the general population. Further, although the subjects appear to have physiological serum cortisol level, some of them may be in pathological condtions such as subclinical Cushing syndrome, and subclinical or partial adrenal insufficiency, since we evaluated the subjects using basal serum cortisol levels alone. However, such subjects may not be so many. Although subclincical Cushing syndrom is much more common than overt Cushing syndrome, its prevalence is not reproted in general population [35]. Since overt Cushing syndrome is a rare disease with an estimated incidence from 0.7 to 2.4 new cases per million population each year [36], the prevalence of subclinical Cushing syndrome also appears to be not substantial. Similarly, prevalence of subclinical or partial adrenal insufficiency is not known in general population, a metaanalysis using the studies with subjects who underwent corticotoropin test for the diagnosis showed that those with basal serum crotisol levels of 5–13 μg/dl were under risk for such insufficiency [37]. Since most subjects of our study have basal cortisol levels in such range (n = 875), a certain number of the subjects appears to have a such insufficiency. However, since we excluded subjects with fasting blood glucose levels below 63 mg/dl, such number may be diminished. Together, the subjects may not accurately represent the general population. In addition, since, as described in the methods, HOMA-R can represent insulin resistance in relatively low fasting blood glucose levels, where effects of GCs on insulin resistnace appear to be limited, the study might not have enough statistical power to evaluate association between GCs and HOMA-R. Further, we used HOMA-ß to evaluate ß-cell function. However, HOMA-ß represents ß-cell function in fasting state, and, thus, does not reflect ß-cell function in response to nutritional stimulation such as glucose stimulated insulin secretion. Finally, as our study is cross-sectional and not a cohort study, we could not assess whether higher serum cortisol levels are a risk for the future incidence of diabetes.

In conclusion, high serum cortisol levels are significantly associated with decreased ß-cell function, even in the physiological cortisol range in a Japanese population. These results suggest that higher serum cortisol levels are a risk factor for future incidence of diabetes, thus further study using a cohort population is warranted.

## Supporting Information

S1 Table Clinical characteristics of the subject according to the gender. (DOCX)
