# Supplementary material for: Association between Higher Serum Cortisol Levels and Decreased Insulin Secretion in a General Population
Source: PLoS One. 2016 Nov 18;11(11):e0166077. doi: 10.1371/journal.pone.0166077 (PMC5115704; doi:10.1371/journal.pone.0166077)
Supplement: S1 Table — (DOCX) [file pone.0166077.s001.docx]

**S1 Table. Clinical characteristics of the subject according to the gender**

|  | | | | |  |
| --- | --- | --- | --- | --- | --- |
| **Characteristics** | **Men** |  | **Women** | **p** | |
| **Number** | **390** |  | **681** | **-** | |
| **Age (yr)** | 52.2±14.9 |  | 55.3±15.1 | 0.001** | |
| **Height (cm)** | 168.7±6.5 |  | 155.0±6.4 | <0.001** | |
| **Body weight (kg)** | 67.3±10.5 |  | 53.2±8.1 | <0.001** | |
| **Body mass index (kg/m^2^)** | 23.60±3.17 |  | 22.17±3.26 | <0.001** | |
| **Fat (%)** | 19.0±5.9 |  | 28.6±7.1 | <0.001** | |
| **Cortisol (µg/dl)** | 10.3±3.5 |  | 8.6±3.3 | <0.001** | |
| **Fasting plasma glucose (mg/dl)** | 81.9±10.5 |  | 80.4±9.7 | 0.013* | |
| **HbA1c (%)** | 5.69±0.40 |  | 5.69±0.34 | 0.986 | |
| **Fasting serum insulin: IRI (µU/ml)** | 4.3±2.4 |  | 4.7±2.5 | 0.02* | |
| **HOMA-R** | 0.88±0.55 |  | 0.93±0.55 | 0.012* | |
| **HOMA-ß** | 113.9±142.8 |  | 134.4±154.2 | <0.001** | |
| **Systolic blood pressure (mmHg)** | 133.3±18.1 |  | 127.7±20.3 | <0.001** | |
| **Diastolic blood pressure (mmHg)** | 80.8±11.1 |  | 76.4±10.9 | <0.001** | |
| **Total cholesterol (mg/dl)** | 198.3±33.8 |  | 202.3±33.3 | 0.066 | |
| **Triglyceride (mg/dl)** | 128.6±115.9 |  | 78.5±40.2 | <0.001** | |
| **HDL cholesterol (mg/dl)** | 59.1±17.0 |  | 69.2±16.1 | <0.001** | |
| **Serum albumin (g/dl)** | 4.55±0.29 |  | 4.47±0.26 | <0.001** | |
| **Serum uric Acid (mg/dl)** | 5.96±1.22 |  | 4.24±0.98 | <0.001** | |
| **Serum urea Nitrogen (mg/dl)** | 15.32±4.29 |  | 14.37±4.18 | <0.001** | |
| **Serum creatinin (mg/dl)** | 0.82 ±0.14 |  | 0.62±0.11 | <0.001** | |
| **BNP (pg/ml)** | 15.8±17.9 |  | 23.9±24.3 | <0.001** | |
| **Adiponectin (mg/dl)** | 8.3±3.8 |  | 13.2±5.9 | <0.001** | |
| **Hypertension: n (%)** | 187(48.0) |  | 279(41.0) | 0.027* | |
| **Hyperlipidemia: n (%)** | 166(42.6) |  | 287(42.1) | 0.894 | |
| **Diabetes: n (%)** | 19(4.9) |  | 24(3.5) | 0.28 | |
| **Drinking alcohol: n (%)** | 287(73.6) |  | 217(31.9) | <0.001** | |
| **Smoking (Never/ Past/ Current):n** | 133/135/121 |  | 552/72/56 | <0.001** | |
|  | | | | |  |

**P<0.05 and <0.01 are indicated by * and **, respectively. Data are mean±SD or number of subjects (%).**
